# Predicting influenza antigenicity from Hemagglutintin sequence data based on a joint random forest method

**DOI:** 10.1038/s41598-017-01699-z

**Published:** 2017-05-08

**Authors:** Yuhua Yao, Xianhong Li, Bo Liao, Li Huang, Pingan He, Fayou Wang, Jiasheng Yang, Hailiang Sun, Yulong Zhao, Jialiang Yang

**Affiliations:** 1 School of Mathematics and Statistics, Hainan Normal University, Haikou, 570100 P. R. China; 2 College of Life Sciences, Zhejiang Sci-Tech University, Hangzhou, 310018 P. R. China; 3 College of Information Science and Engineering, Hunan University, Changsha, 410082 P. R. China; 4 College of Sciences, Zhejiang Sci-Tech University, Hangzhou, 310018 P. R. China; 5 School of Mathematics and Information Science, Henan Polytechnic University, Henan, 454000 P. R. China; 6 Department of Civil and Environmental Engineering, National Universality of Singapore, Singapore, 119077 Singapore; 7 College of Veterinary Medicine, Huanan Agricultural University, Guangzhou, 510000 P. R. China; 8 Department of Mathematics, City University of Hong Kong, Hong Kong, P. R. China; 9 Department of Genetics and Genomic Sciences, Icahn School of Medicine at Mount Sinai, NY, 10029 USA

## Abstract

Timely identification of emerging antigenic variants is critical to influenza vaccine design. The accuracy of a sequence-based antigenic prediction method relies on the choice of amino acids substitution matrices. In this study, we first compared a comprehensive 95 substitution matrices reflecting various amino acids properties in predicting the antigenicity of influenza viruses by a random forest model. We then proposed a novel algorithm called joint random forest regression (JRFR) to jointly consider top substitution matrices. We applied JRFR to human H3N2 seasonal influenza data from 1968 to 2003. A 10-fold cross-validation shows that JRFR outperforms other popular methods in predicting antigenic variants. In addition, our results suggest that structure features are most relevant to influenza antigenicity. By restricting the analysis to data involving two adjacent antigenic clusters, we inferred a few key amino acids mutation driving the 11 historical antigenic drift events, pointing to experimentally validated mutations. Finally, we constructed an antigenic cartography of all H3N2 viruses with hemagglutinin (the glycoprotein on the surface of the influenza virus responsible for its binding to host cells) sequence available from NCBI flu database, and showed an overall correspondence and local inconsistency between genetic and antigenic evolution of H3N2 influenza viruses.

## Introduction

Causing an estimated 500,000 deaths worldwide per year, influenza epidemics in humans seriously endanger population health and world economy^1^. Vaccination is the primary option to reduce influenza outbreaks. The efficacy of a seasonal influenza vaccine depends largely on the selection of vaccine strains, i.e., the strains this vaccine is designed to prevent. A good vaccine recipe should target potential circulating strains able to escape from population immunity in the new flu season^2^. However, influenza viruses are classic examples of antigenically variable pathogens and have a seemingly endless capacity to evade immune response^3^, which makes vaccine design extremely challenging. According to reports from the center for disease control and prevention (CDC), flu shots fail half of the time^4^. As such, timely identification of emerging antigenic variants is critical to influenza vaccine design, flu surveillance, and human health^5^.

One of the most popular assays to evaluate the efficacy of a vaccine against an influenza virus is the hemagglutination inhibition (HI) assay, a binding assay measuring the ability of antisera (vaccine) to block the hemagglutinin (HA) of the antigen (virus) from agglutinating red blood cells^6^. However, HI assay is labor and cost intensive, which poses the need for efficient computational methods to estimate the antigenic similarity between antigens and antisera^2^. With the advances of sequencing techniques, influenza sequences have become more and more available^7^, making them good candidates for predicting the antigenicity of new viruses and identifying antigenic variants.

Popular antigenic prediction methods include imputation-based methods^5, 8^, and sequence-based methods. Sequence-based methods usually associate mutations in HA proteins with antigenic differences (among viruses) obtained from serological tests^5, 8, 9^. The antigenic differences are either quantified by antigenic distances^5^ or simply represented by a binary value to indicate if two viruses are antigenic variants^9, 10^. For example, Liao *et al*. tested four algorithms including iterative filtering, multiple regression, logistic regression, and support vector machine to predict antigenic variants from mutations in HA1, a sub-unit of HA forming globular domain^11^. They also explored six amino acids substitution models based on physiochemical grouping of 20 amino acids^10^. Sun *et al*. proposed Antigen-Bridges, a bootstrapped ridge regression model, to predict antigenic distances using amino acids substitutions quantified by pattern-induced multisequence alignment (PIMA) in HA1 protein sequences^2^. As H3N2 influenza viruses from 1968 to 2003 are grouped into 11 antigenic clusters, i.e, HK68, EN72, VI75, TX77, BK79, SI87, BE89, BE92, WU95, SY97, and FU02 in chronological order^5^, they also predicted HA mutations driving antigenic drift events between adjacent clusters and experimentally validated two predicted mutation sets (i.e., from BE92 to WU95 and from WU95 to SY97). Noticing that co-evolution in HA1 might contribute to antigenic evolution, Yang *et al*. developed a Lasso model incorporating both single and co-mutation features in HA1 protein sequences^12^. More recently, Qiu *et al*. developed a protein structure-based antigenic prediction model^13^, and Neher *et al*. developed an optimization model to interpret known antigenic data and evaluated its ability in predicting composition of future influenza virus populations^14^. There are also many other methods in this hot topic, e.g., Huang *et al*.^15^ and Ren *et al*.^16^ to name but a few.

Despite the fact that these methods are greatly helpful in selecting antigenic variants and optimizing vaccine strains, there are still a few points to be improved. First, it is known that the scoring matrices to quantify amino acids substitutions are critical to the accuracy of prediction algorithms^10, 13^. However, only a few matrices reflecting partial protein attributes have been tested, e.g., binary substitution matrix^17^, physiochemical models^10^, PAM250, BLOSUM62, PIMA^12^, and structure model^13^. A systematic study of the relationship between amino acids attributes and influenza antigenicity is largely missing. AAindex, a database of numerical indices representing various physicochemical and biochemical properties of amino acids and pairs of amino acids, provides an opportunity to fix this gap^18^. The predictive powers of the 94 physicochemical and biochemical properties of amino acids in AAindex could be helpful to elucidate their contribution to influenza antigenic evolution. Second, it is unclear if the combination of a few important amino acids attributes can better predict influenza antigenicity. Third, most previous models either predict antigenic variants or adopt linear models. There might be some advantages in predicting continuous antigenic distances using nonlinear models like random forest^19^ since antigenic distances have higher resolution than binary values and the relationship among antigenic sites might be nonlinear.

In this study, we propose and test Joint Random Forest Regression (JRFR), a novel algorithm that combines multiple substitution matrices into the random forest algorithm to predict antigenic distances from HA1 protein sequences. We also systematically compare 95 amino acids substitution matrices in predicting the antigenicity of H3N2 influenza viruses. These substitution matrices reflect a comprehensive list of amino acids attributes including structural, physicochemical, and biochemical information. Finally, we explore the relationship between genetic and antigenic evolution of H3N2 influenza viruses based on the prediction results from JRFR.

## Results

### JRFR: a random forest model to predict antigenic distances

**Figure 1:**
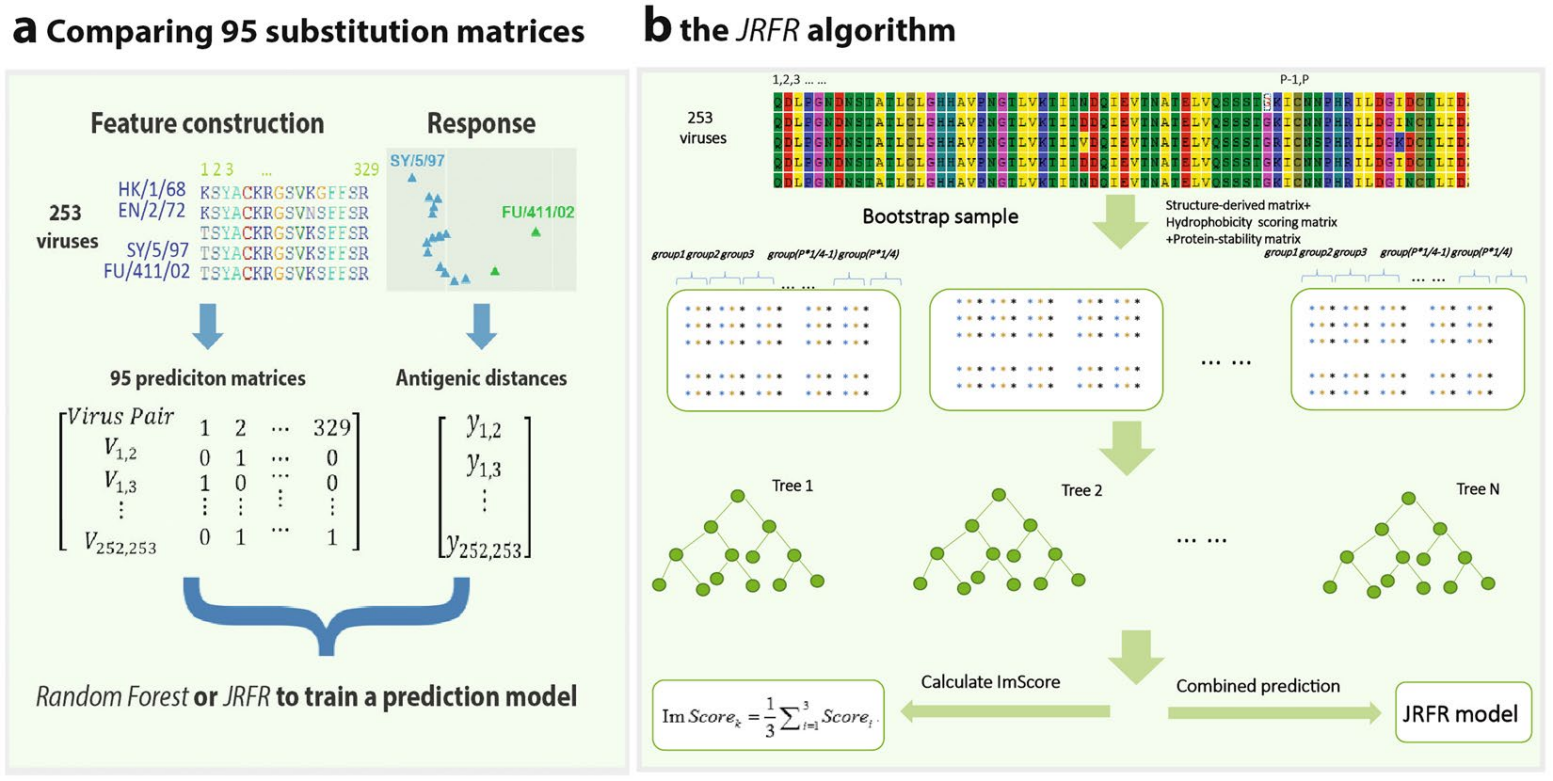
A flowchart to illustrate the computational framework in this study.

We illustrated our computational framework in Fig. 1. We trained a predictive model by taking the amino acids’ changes among virus pairs at each protein site as a feature and the pairwise antigenic distances among viruses as the response (see Materials and Methods for details). There are many amino acids substitution matrices reflecting different attributes of amino acids^18^ and the choice of substitution model has been proven to be critical in prediction accuracy^9, 10, 16^. Thus, we first evaluated the prediction powers of a comprehensive 95 amino acids substitution matrices (i.e, 94 matrices in AAindex^18^ and binary substitution one) by applying the random forest algorithm (Fig. 1a). We then proposed the JRFR algorithm by jointly considering 2 or more substitution matrices. Specifically, we first selected the top 15 substitution matrices according to 10-fold cross-validation prediction accuracy, each was considered as a major matrix. The 94 secondary matrices (matrices other than the major matrix) were then selected one by one to join the model in a greedy manor to improve prediction accuracy (see Materials and Methods). The top model was selected for predicting the antigenicity of new influenza viruses. For simplicity, we only allowed at most 2 secondary matrices. Based on the importance score of each protein locus in JRFR, we also evaluated the contribution of a mutation to antigenic change and antigenic drift events.

### Evaluation of amino acids attributes in predicting influenza antigenicity

We applied our computational framework into the H3N2 human influenza data from 1968 to 2003^5^, a curated HI table of 253 viruses and 79 vaccines. According to Smith *et al*.^5^ the viruses are classified into 11 antigenic clusters namely HK68, EN72, VI75, TX77, BK79, SI87, BE89, BE92, WU95, SY97, and FU02, respectively. The pairwise antigenic distances among the viruses were calculated based on Metric MDS^5^ and were used as responses in our models. We then downloaded the HA1 protein sequences (of length 329) for the 253 viruses from NCBI flu database^7^, and aligned them using MUSCLE^20^. The alignment was then transformed into 95 feature matrices based on the 94 amino acids substitution models in AAindex^18^ and the binary substitution model. The 10-fold cross-validation root-mean-square errors (RMSEs) for all 95 models were summarized in Supplementary Table S1, among which we listed a few top or well studied models in Table 1.

**Table 1:** The top 12 amino acids substitution matrices in predicting influenza antigenicity.

Accession No	Description	RMSE
NIEK910102	Structure-derived correlation matrix 2	0.965
NIEK910101	Structure-derived correlation matrix 1	0.967
RIER950101	Hydrophobicity scoring matrix	0.968
MIYS930101	Base-substitution-protein-stability matrix	0.968
DOSZ010102	Normalised version of SM_SAUSAGE	0.970
LUTR910102	Structure-based comparison table for inside other class	0.970
AZAE970102	The substitution matrix derived from spatially conserved motifs	0.971
BENS940101	Log-odds scoring matrix collected in 6.4–8.7 PAM	0.972
TUDE900101	isomorphicity of replacements	0.973
AZAE970101	The single residue substitution matrix from interchanges of spatially neighbouring residues	0.973
HENS920102	BLOSUM62 substitution matrix	0.986
DAYM780301	Log odds matrix for 250 PAMs	1.000
Binary	1 for substitution and 0 for match	1.098

As can be seen, the selection of substitution models indeed has some influences in prediction accuracy. The best model “Structure-derived correlation matrix 2” has a RMSE 0.965, 15% lower than that of the worst model “Context-dependent optimal substitution matrices for buried coil” (1.111). In Cai *et al*.^17^ it was shown that the 10-fold cross-validation RMSEs are 1.051 for their MC-MDS model and 1.047 for Metric MDS model^5^ using the same data. So our best model has 8.5% lower RMSE than both models. Interestingly, the top models are derived from structure-based substitution matrices, implying the importance of HA1 protein structure in influenza antigenicity. It is quite reasonable since antigenicity measures the binding affinity between antigen and antiserum, in which structure information is critical^6^. A few commonly used substitution models including binary (1.098), PAM250 (0.977), and BLOSUM62 (0.986) perform not as good as structure ones. The reason might be that they could not reflect much information related to antigen-antiserum reaction.

Since influenza antigenicity is known to associate with many attributes of HA protein, e.g., structure and hydrophobicity, we conjectured that it might be helpful to combine a few amino acids characteristics. Thus, we also applied our JRFR model by combining multiple substitution matrices. The prediction accuracy is indeed improved by JRFR (see Table 2 for a few top combinations), confirming that influenza antigenicity is affected by many protein attributes. The best combination uses NIEK910101 mutation matrix (“Structure-derived correlation matrix 1”) as the main feature matrix and RUSR970101 (“Substitution matrix based on structural alignments of analogous proteins”) and KOSJ950107 (“Context-dependent optimal substitution matrices for buried turn”) as auxiliary feature matrices. The 10-fold cross-validation RMSE could be reduced to 0.941 (with prediction accuracy 0.963, sensitivity 0.980, specificity 0.775 and the Mathew’s correlation coefficient (MCC) 0.756) by this model. It is of note that the prediction accuracy could be further improved by adding new secondary matrices, however, the improvement is marginal. As such, we adopt the best model with 3 feature matrices for all subsequent analyses.

**Table 2:** Performances of seven prediction models.

Main data	Merge data 1	Merge data 2	RMSE	Accuracy	Specificity	Sensitivity	MCC
NIEK910101	RUSR970101	KOSJ950107	0.941	0.963	0.775	0.980	0.756
RIER950101	FEND850101	FEND850101	0.943	0.964	0.777	0.981	0.758
NIEK910101	RUSR970101	None	0.950	0.963	0.767	0.981	0.753
RIER950101	FEND850101	None	0.952	0.963	0.771	0.980	0.753
NIEK910102	RUSR970101	None	0.952	0.963	0.767	0.981	0.754

### Relationship between influenza genetic and antigenic evolution as revealed by JRFR

**Figure 2:**
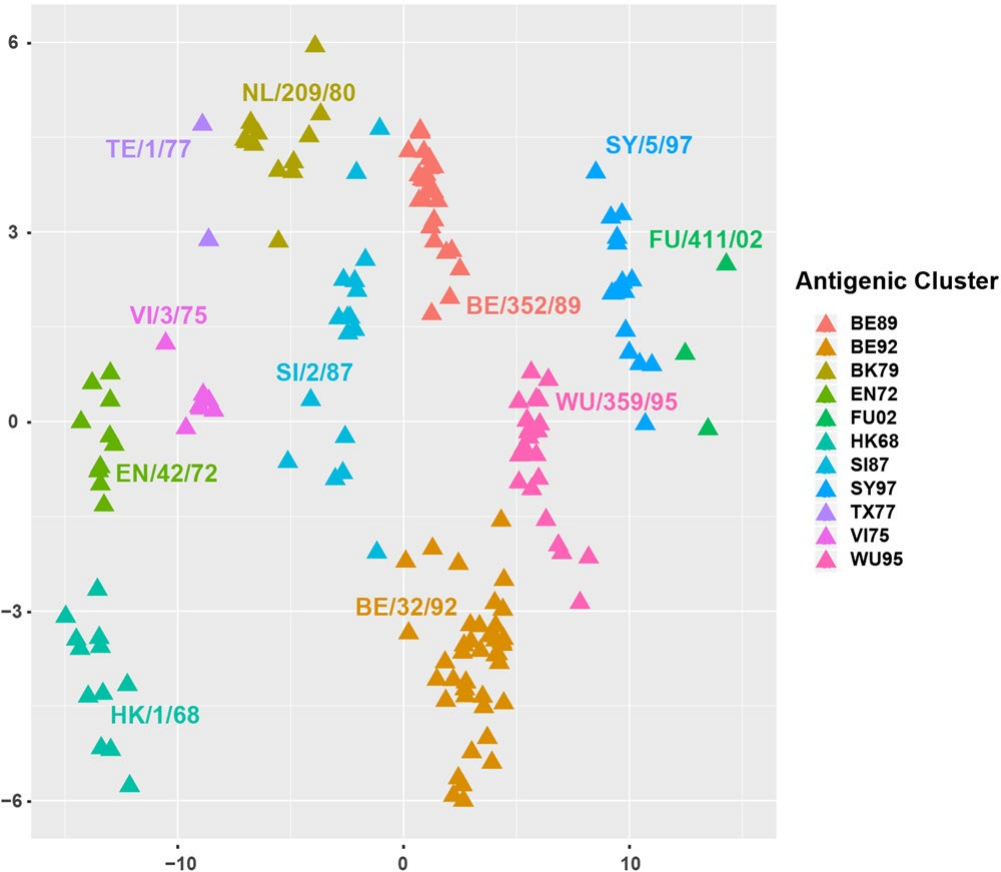
The antigenic map of 253 H3N2 influenza viruses predicted by JRFR. The 11 antigenic clusters HK68, EN72, VI75, TX77, BK79, SI87, BE89, BE92, WU95, SY97, and FU02 are marked by different colors.

Based on the antigenic distances inferred by JRFR, we constructed the antigenic cartography of the 253 H3N2 viruses by multidimensional scaling (see Fig. 2). As can be seen from the figure, all the 11 antigenic clusters are well separated. The viruses have been evolving alongside an S-shaped path antigenically. Viruses in a few clusters are more compact (e.g., BE89) while others are more spread (e.g., SI87). Sequence-based antigenic cartography could be useful in selecting potential antigenic variants for further serological test and thus benefits vaccine design^2^.

**Figure 3:**
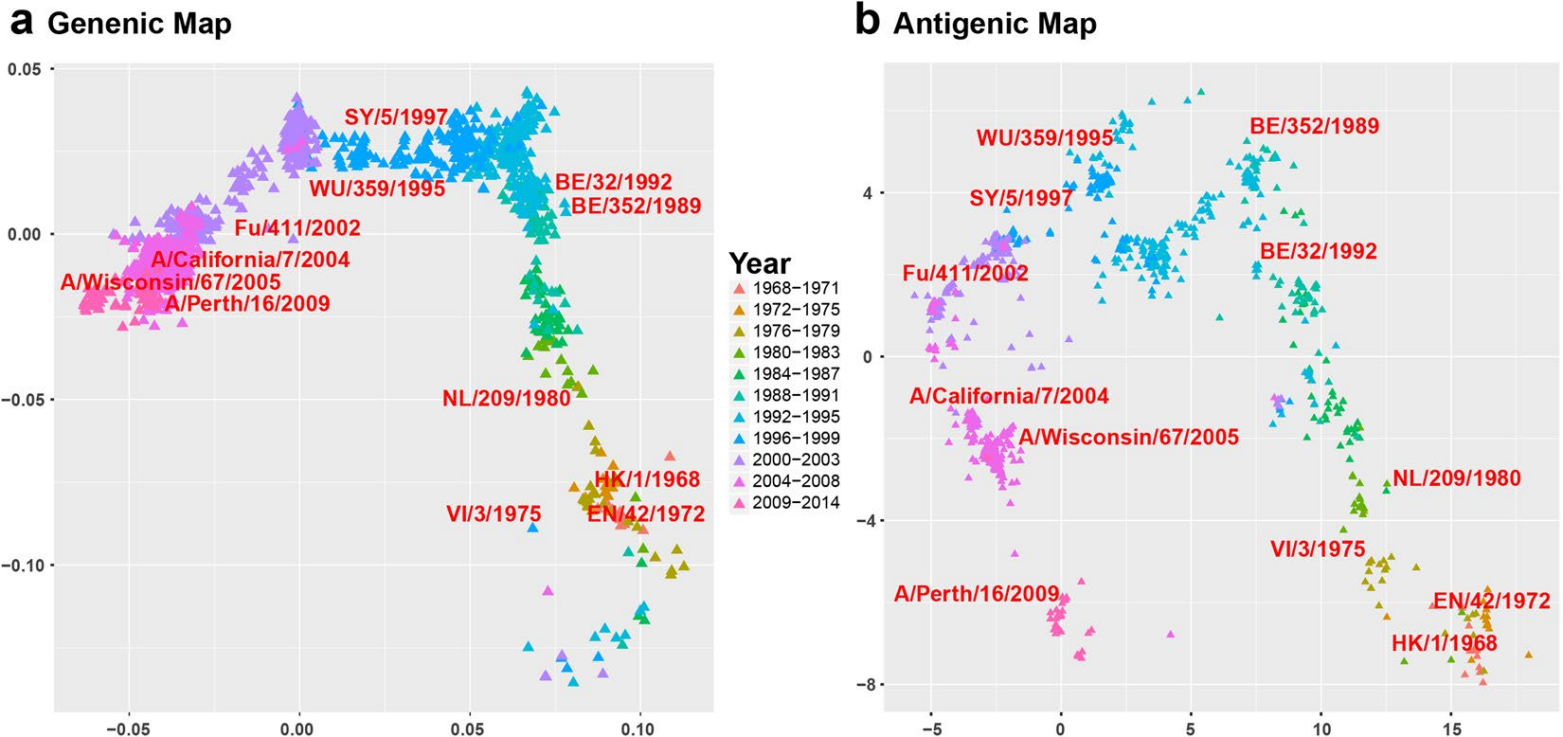
The genetic (**a**) and antigenic map (**b**) for all 1968 non-redundant H3N2 HA1 protein sequences between 1968 and 2014. The viruses are colored by their year of discovery.

Since 253 viruses might only represent partial antigenic evolution, we downloaded all 1638 non-redundant H3N2 HA1 protein sequences between 1968 and 2014 from NCBI flu database and predicted their antigenic distance using JRFR. We plotted their genetic and antigenic map (see Materials and Methods) in Fig. 3. As can be seen, the genetic and antigenic maps are generally consistent. However, the genetic map is more continuous while the antigenic map is more punctual. The result is consistent with a few previous studies^2, 5^. To evaluate historical vaccine strains, We marked a few known vaccine strains in both genetic and antigenic maps. We found that some vaccine trains are very close to each other in the genetic map but relative far in the antigenic map. For example, the genetic distance between BE/352/1989 and BE/32/1992 is less than 0.01, however their antigenic distance is larger than 3. Similar scenarios could be found for A/Wisconsin/67/2005 and A/Perth/16/2009. The observation indicates that the contribution of genetic mutation to antigenicity is different at different protein sites. Only a small set of sites might be responsible for antigenic evolution^5^.

### Sites driving antigenic changes in H3N2 influenza A viruses

**Figure 4:**
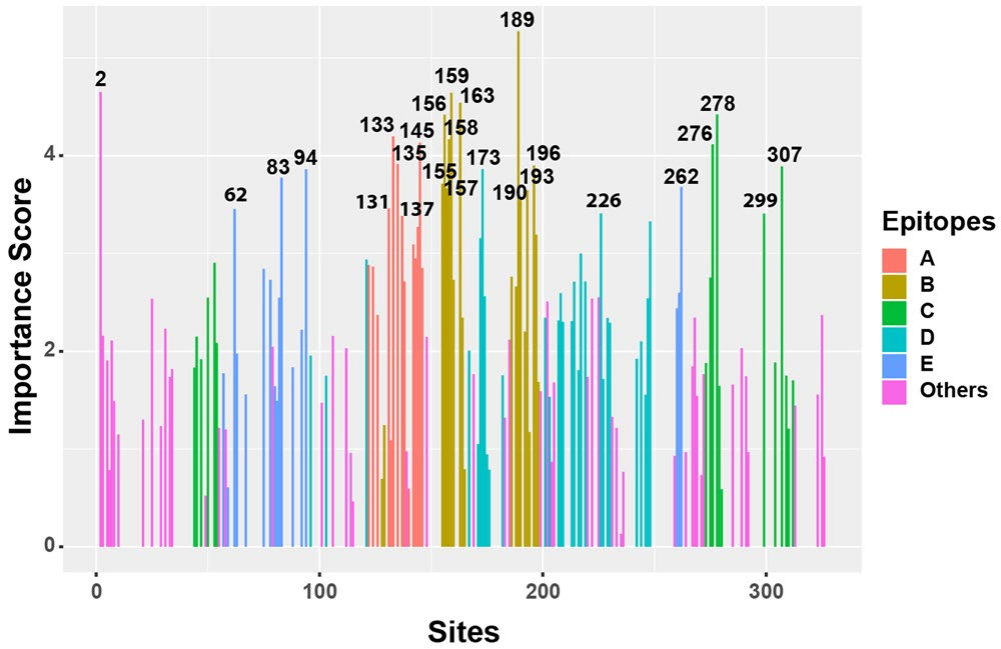
The importance score of all 329 sites predicted by JRFR. The 5 epitopes A, B, C, D, and E are marked in different colors. The remaining sites are classified as “Others”.

We inferred the antigenic importance of each protein sites by their importance score in the JRFR model. We plotted the log10-transformed importance score for all 329 sites in HA1 protein in Fig. 4 and listed the actual values of the top 34 sites (with log10 importance large than 2.9) in Table 3. It is known that HA consists of five epitopes (epitope A, B, C, D, and E), each having around 20 structural neighbour amino acids locating on the protein surface^21–23^. A few recent studies experimentally and computationally identified important antigenic sites, most of which locate on the 5 epitopes^2, 12, 24, 25^. As can be seen from Table 3, vast majority of the key sites are located on the five epitopes^21–23^ especially on epitope A and B. Among the top 34 antigenically important sites, there are 11 in epitope B (i.e, 189, 163, 159, 156, 158, 196, 190, 155, 193, 157, and 197), 8 in A (i.e., 133, 145, 135, 137, 131, 144, 143, and 142), 6 in D (i.e., 173, 226, 248, 121, 217, and 172), 5 in C (i.e., 278, 276, 307, 53, and 299), and 4 in E (i.e., 83, 94, 262, and 62). Interestingly, we also identified an antigenic sites (i.e., 2) not belonging to any epitope, which is consistent with a few previous studies^2, 12^. These sites might be important in driving future antigenic evolution of H3N2 influenza A viruses.

**Table 3:** The top 34 antigenic importance sites according to importance scoring in JRFR for H3N2 influenza data.

Site	Importance Score	Antigenic Domain	Site	Importance Score	Antigenic Domain
189	5.273	B	262	3.678	E
2	4.651	other	157	3.664	B
159	4.645	B	193	3.646	B
163	4.541	B	190	3.545	B
278	4.421	C	131	3.460	A
156	4.417	B	62	3.453	E
133	4.196	A	226	3.408	D
158	4.167	B	299	3.406	C
145	4.133	A	137	3.383	A
276	4.116	C	248	3.326	D
135	3.915	A	144	3.270	A
196	3.899	B	197	3.189	B
307	3.890	C	172	3.154	D
173	3.862	D	142	3.091	A
94	3.859	E	217	2.998	D
83	3.775	E	143	2.946	A
155	3.712	B	121	2.936	D

### Mutations driving H3N2 influenza antigenic drift events

There are 10 antigenic drift events for H3N2 influenza viruses occurred between 1968 and 2003^5^. We also applied JRFR on viruses in chronologically adjacent antigenic clusters to infer amino acids mutations driving specific drift event based on importance scores. The combination of mutations responsible for the 10 drift events were listed in Table 4. Interestingly, we observed a few antigenic sites critical for multiple antigenic drift events. For example, site 193 drove the early two antigenic drift events, i.e., HK68-EN72 and EN72-VI75. Site 145 was involved in 4 antigenic drifts, i.e., EN72-EN75, SI87-BE89, BE89-BE92, and BE92-WU95.

**Table 4:** Multiple mutations driving 10 antigenic drifts for H3N2 influenza viruses inferred by JRFR.

Antigenic drift events	Combination of mutations
HK68-EN72	S193N-G144D
EN72-VI75	N53D-S193D-S145N
VI75-TX77	S137G
TX77-BK79	D144V-N2K
BK79-SI87	Y155H-K189R
SI87-BE89	N145K, N145K-G135E, N145K-N193S
BE89-BE92	K145N-E156K-R189S, K145N-E156K-T262N-R189S, K145N-E156K-S133D-R189S
BE92-WU95	K135T-N145K-L226V, K135T-N145K-N262S
WU95-SY97	V196A-N276K-E158K-K156Q, V196A-N276K-E158K-K156Q-K62E
SY97-FU02	A131T-H155T-V202I

**Figure 5:**
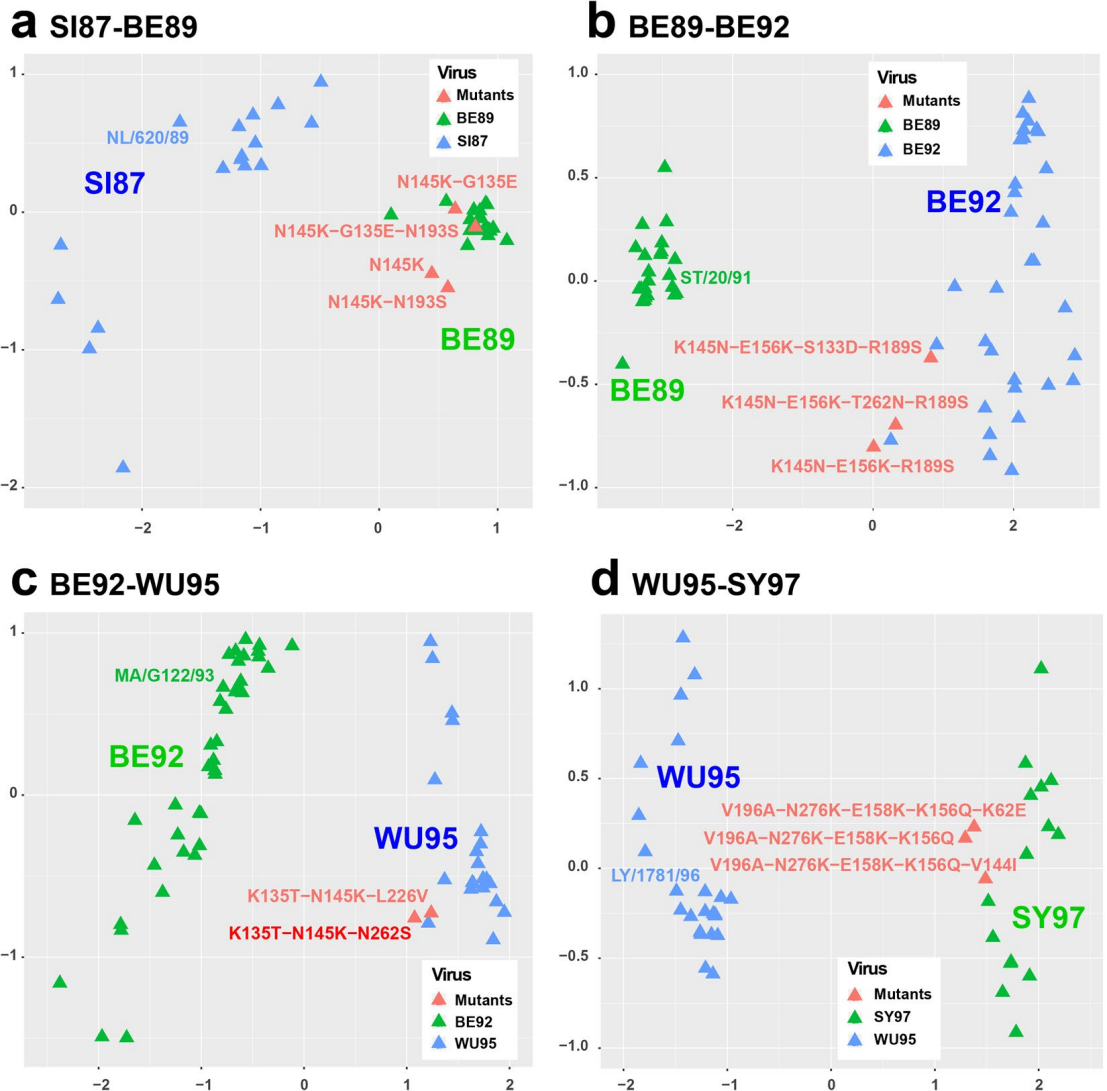
Antigenic cartographies to illustrate the key mutations driving the 4 antigenic drift events including (**a**) SI87-BE89, (**b**) BE89-BE92, (**c**) BE92-WU95, and (**d**) WU95-SY97.

To validate the power of these sites in driving antigenic drift events, we manually selected one virus at the center of one antigenic group, and mutated its amino acids, by which we obtained a few artificial variants of the selected virus. We then predicted the antigenicity of the variants (based on its HA1 sequence) and test if it has similar antigenicity with viruses in the later antigenic group. For a better view, we plotted the antigenic cartography of the viruses in two adjacent antigenic clusters, the selected virus, and its artificial variants (see Fig. 5). We only plotted the late 4 clusters since they have more viruses. As one can see, in most cases the predicted mutation combinations are capable of driving the antigenic drifts. Sun *et al*. experimentally validated that N145K can drive BE92-WU95, which is consistent with our predictions^2^.

## Discussion

As we know, the antigenicity of influenza viruses changes rapidly and vaccines should be updated accordingly to avoid influenza outbreaks. The vaccines have been updated at least 27 times for H3N2 viruses since 1968, 9 times for H1N1 virus from 1977 to 2009, and 15 times for influenza B virus from 1972 to 2011^2^. However, the selection of vaccine strains is non-trivial due to the exceptional large number of new influenza viruses in the new season and the burden in testing antigenicity of these virus against known vaccines. As a result, flu shots from CDC fail half the time^4^. Thus, timely surveillance of the antigenic evolution of these viruses is critical. However, traditional experimental methods such as HI assay for evaluating the antigenicity of new viruses face a multitude of issues. For example, the reduction seen in H3N2 virus binding to red blood cells^26, 27^ can lead to problems performing and interpreting HI assays. Furthermore, because antigenic characterization is relatively labor-intensive, only a small portion (generally, fewer than 20%) of the influenza isolates sequenced will be antigenically characterized.

With the development of sequencing techniques, influenza protein sequencing has become a routine for influenza studies. Many newly sequenced influenza protein sequences have been stored in influenza databases like NCBI influenza database^15^. Much of the burden for influenza surveillance could be avoided if reliable sequence-based antigenic prediction methods can be established. JRFR produces relative low prediction error and reasonable antigenic cartography, which could serve as an initial screen of antigenic variants for further experimental analyses.

In the JRFR framework, we systematically evaluated the powers of 95 amino acids substitution matrices in predicting influenza antigenicity, which reflecting various physicochemical and biochemical properties of amino acids. We found that structure-based features outperformed all other features, followed by amino acids hydrophobicity. The importance of HA structure in influenza antigenicity has long been identified^28^. Interestingly, commonly used substitution matrices including binary matrix, PAM250 and BLOSUM62 are not performing well, suggesting the necessity of combining protein structure information in studies to predict phenotypes using protein sequences. By combining a few feature matrices, the prediction accuracy could be further improved, indicating that influenza antigenicity might be determined by multiple factors, though HA structure might play dominant roles.

In addition, we inferred 34 antigenically important protein sites, most of which are located at the 5 epitopes, especially in epitope A and B. Ndifon *et al*.^29^ presented a competitive model to predict antibody escape and proposed that antigenic drift events would be associated with amino acid changes that occur in epitopes with high neutralization efficiencies (i.e., epitopes A, B, and D) rather than in those with low neutralization efficiencies (i.e., epitopes C and E). The results are consistent with our findings. Meanwhile, we found that a small subset of amino acids mutations could drive most antigenic drift events for H3N2 influenza viruses, confirming the result from a few previous studies^2, 12^.

In the end, we would like to point out that our framework works generally for all influenza sub-types though we used H3N2 influenza as an illustration. In the future, we plan to predict the antigenicity of other sub-types like H1N1, H5N1 and H7N9, and compare their antigenically associated amino acid mutations or antigenic determinant regions. In addition, our method could also be applied to investigate other problems, such as disease and drug prediction, DNA-binding protein prediction^30^, protein fold recognition^31^, detection of tubule boundary^32^, and other related problems^33, 34^ etc. However, it is out of the scope of this study.

## Materials and Methods

### Influenza data

In this study, we adopted the H3N2 influenza data from Smith *et al*.^5^ which contains a partially revealed HI table consisting of 253 viruses and 79 vaccines from 1968 to 2003. There are 11 major antigenic clusters including HK68, EN72, VI75, TX77, BK79, SI87, BE89, BE92, WU95, SY97 and FU02, named by vaccine strain in the respective cluster^5^. We also downloaded the HA protein sequences of the 253 viruses and those of a total 1638 viruses available from NCBI flu database from 1968 to 2014. The HA protein sequences were aligned by MUSCLE^20^, and we only kept the 329 sites belong to HA1 protein for further analyses.

### The random forest algorithm

We applied the random forest algorithm to predict antigenic distances using 95 single amino acids substitution models^35^. It is a nonlinear ensemble algorithm taking each non-conservative protein site as a feature and pair-wise antigenic distances among viruses as responses. Let *n* be the number of viruses (*n* = 253 for H3N2 data^5^) and **S** be the alignment of their HA1 sequences. Since conservative sites are non-informative, we removed them from **S**. Let *m* be the number of non-conservative sites (*m* = 154 for H3N2 data). Thus after removing conservative sites, **S** is an *n* × *m* (253 × 154 for H3N2 data) matrix with each entry being an amino acid.

#### Feature construction

We constructed the feature matrix **X** as follows: Let **S**_*ij*_ be the amino acid at position (*i*, *j*) in **S** and **X**_*j*_ be the *j* ^*th*^ column of **X**. Then ${{\bf{X}}}_{j}=\{{D}_{{S}_{kj},{S}_{lj}}\}$, a vector traversing all ordered pairs of (*k*, *l*) with 1 ≤ *k* ≤ *l* ≤ *n*. Clearly, **X**_*j*_ is a vector of length $$N=(\begin{array}{c}n\\ 2\end{array})$$ and **X** is an *N* × *m* matrix. Here, **D**_*a,b*_ denotes the dissimilarity between amino acids *a* and *b*. It is of note that AAindex only provides similarity matrix **A** among amino acids. We transformed **A** into dissimilarity matrix **D** using the following formula,

1 $${{\bf{D}}}_{a,b}=({{\bf{A}}}_{a,a}+{{\bf{A}}}_{b,b})-2{{\bf{A}}}_{a,b}.$$

#### Response

We adopted antigenic distance proposed by Smith *et al*.^5^ as our response **y**, where **y** consists of all distances between ordered pairs of viruses. Clearly, **y** is also of length *N*. Given an HI table of *n* viruses, **y** could be calculated directly from the website provided in ref. 5.

After the feature matrix **X** and the response vector **y** were constructed, we applied the random forest function in R package ‘randomForest’ to construct the prediction model^36^. We set the bootstrapping (tree) number to be 500 and the number of features to be $\frac{m}{4}$ for each tree. We then tested and compared the 95 dissimilarity matrices based on a 10-fold cross-validation process and used root-mean-square error (RMSE) to evaluate their performances, which are described as follows.

#### RMSE

Let *α* = (*α*_1_, *α*_2_, …, *α*_*N*_) and *β* = (*β*_1_, *β*_2_, …, *β*_*N*_) be two prediction vectors, then the RMSE between *α* and *β* is defined as

2 $$RMSE(\alpha ,\beta )=\sqrt{\frac{\sum _{i=1}^{N}{({\alpha }_{i}-{\beta }_{i})}^{2}}{N}}$$

#### Accuracy and specificity

For a pair of viruses with underlying antigenic distance *y*, we define them to be true antigenic variants if *y* ≥ 2, and false otherwise. Similar they are called positive antigenic variants if the predicted antigenic distance *ỹ* ≥ 2 by JRFR, and negative otherwise. By this way, we can define true positive (TP) pairs (i.e., *y* ≥ 2 and *ỹ* ≥ 2), false positive (FP) (*y* < 2 and *ỹ* ≥ 2), true negative (TN) (*y* < 2 and *ỹ* < 2), and false negative (FN) (*y* ≥ 2 and *ỹ* < 2). The accuracy of a method is defined as

3 $$accuracy=\frac{TP+TN}{TP+FP+TN+FN}$$

and the specificity is defined as

4 $$specificity=\frac{TN}{TN+FP}.$$

#### 10-fold cross-validation

We divided **y** randomly into 10 equal parts, 9 parts of which (with the corresponding sub-matrices of **X**) are used for training and the remaining one part for prediction. The process is repeated for 10 times until each part is used as the prediction set once. By merging the prediction results for each part, we obtained a prediction vector $\tilde{{\bf{y}}}$. The RMSE between **y** and $\tilde{{\bf{y}}}$ was used to tune model parameters and compare the performances of different models.

### The JRFR algorithm

In the JRFR algorithm, we combined multiple feature matrices (derived from different amino acid similarity matrices in AAIndex^18^) to construct decision trees in random forest algorithm. Specifically, let **X** ^(1)^, **X** ^(2)^, …, **X** ^(*k*)^ be *k* feature matrices, each consisting of *m* features corresponding to *m* non-conservative sites in HA1. We set one of the feature matrices, say **X** ^(1)^ to be the main matrix and other to be auxiliary ones. For each auxiliary matrix **X** ^(*i*)^ with 2 ≤ *i* ≤ *k*, we then applied an linear regression process to remove its overlapping information from the main matrix as follows: let ${{\bf{X}}}_{j}^{(i)}$ and ${{\bf{X}}}_{j}^{\mathrm{(1)}}$ be the *j* ^*th*^ feature vectors of **X** ^(*i*)^ and **X** ^(1)^ respectively. There exist constants *α*_*ij*_, *β*_*ij*_, and vector *ε*_*ij*_ such that

5 $${{\bf{X}}}_{j}^{(i)}={\alpha }_{ij}+{\beta }_{ij}{{\bf{X}}}_{j}^{\mathrm{(1)}}+{\varepsilon }_{ij}$$

where *α*_*ij*_ is the intercept, *β*_*ij*_ is the regression coefficient and *ε*_*ij*_ is the residual vector. We constructed a new feature matrix with each feature vector being *ε*_*ij*_ for 1 ≤ *j* ≤ *m*. For brevity, we still used **X** ^(*i*)^ to represent this matrix for 2 ≤ *i* ≤ *k*. By this way, we obtained new *k* − 1 auxiliary matrices independent of **X** ^(1)^.

We then combined the main feature matrix and new auxiliary ones to predict antigenic distance using random forest algorithm. That is, we extended the number of features to *k* × *m*, and applied the usual random forest process introduced above. In practice, we only used the top 15 feature matrices (as ranked in Supplementary Table S1) as main feature matrices and restricted the number of auxiliary feature matrices to be less than 2. Similar to single feature matrix analysis, we set the bootstrapping (tree) number to be 500 and the number of features to be $\frac{k\,\ast \,m}{4}$ for each tree.

#### Importance score

To calculate the importance of a feature, the random forest algorithm permutes the values of this feature. It then calculates and normalizes the difference of out-of-bag error before and after permutation^35^. By applying the random forest function in **R**, we obtained importance scores for features in both main feature matrix and auxiliary ones. Let ${{\bf{I}}}_{j}^{(i)}$ be the importance score of the *j* ^*th*^ feature in the main feature matrix and ${{\bf{I}}}_{j}^{(i)}$ with 2 ≤ *i* ≤ *k* be its importance scores in auxiliary feature matrices. Since the *j* ^*th*^ feature in both main matrix and auxiliary ones represents information from the *j* ^*th*^ non-conservative site in HA1 sequence, we defined the importance of the *j* ^*th*^ non-conservative site to be $\frac{{\sum }_{i=1}^{k}{{\bf{I}}}_{j}^{(i)}}{k}$.

#### Building the JRFR prediction model

We sorted all non-conservative sites in descending order based on their importance scores, and selected site one by one from top to join the JRFR algorithm, until the best 10-fold cross-validation RMSE is obtained. For H3N2 data, we selected the top 85 sites to building the final JRFR model by this process.

### Construction of antigenic and genetic cartography

The antigenic map was directly constructed from the predicted antigenic distance matrix among viruses using classical multidimensional scaling (MDS). To construct the genetic map, we first calculated the p-distance matrix between pair of viruses and then applied classical MDS.

## Electronic supplementary material

Supplementary Table S1
